# Medical image translation using an edge-guided generative adversarial network with global-to-local feature fusion

**DOI:** 10.7555/JBR.36.20220037

**Published:** 2022-06-28

**Authors:** Hamed Amini Amirkolaee, Hamid Amini Amirkolaee

**Affiliations:** 1 School of Surveying and Geospatial Engineering, College of Engineering, University of Tehran, Tehran 1417935840, Iran; 2 Civil and Geomatics Engineering Faculty, Tafresh State University, Tafresh 7961139518, Iran

**Keywords:** edge-guided generative adversarial network, global to local, medical image translation, magnetic resonance imaging, computed tomography

## Abstract

In this paper, we propose a framework based deep learning for medical image translation using paired and unpaired training data. Initially, a deep neural network with an encoder-decoder structure is proposed for image-to-image translation using paired training data. A multi-scale context aggregation approach is then used to extract various features from different levels of encoding, which are used during the corresponding network decoding stage. At this point, we further propose an edge-guided generative adversarial network for image-to-image translation based on unpaired training data. An edge constraint loss function is used to improve network performance in tissue boundaries. To analyze framework performance, we conducted five different medical image translation tasks. The assessment demonstrates that the proposed deep learning framework brings significant improvement beyond state-of-the-arts.

## Introduction

Medical imaging is a very important technique for diagnosing and when treating numerous diseases. Generally speaking, using a single imaging modality is insufficient in clinical decision-making and it is necessary to combine different modalities. Each modality has unique features with varying degrees of sensitivity and specificity, therefore set of images is required for accurate and reliable clinical decisions. A desirable approach for many diagnostic and therapeutic purposes is to generate synthesized hypotheses using medical images and image translation such as computed tomography (CT) to magnetic resonance imaging (MRI).

The traditional approach to image translation can be categorized as either atlas-based, tissue-segmentation-based, or learning-based approaches^[1]^. In atlas-based approaches, an image registration method is applied to align an MRI to an atlas MRI for approximating a correspondence matrix. The approximated matrix can be used to estimate query MRIs by warping the associated atlas CT image^[2–4]^. These approaches require an accurate deformable image registration of the atlas and patient MRIs. In tissue-segmentation-based approaches, MRI voxels are segmented into different tissue classes, *i.e.*, soft tissue, fat, bone, and air. Then, class segments are refined manually^[5–6]^; however, using only one MRI is not sufficient to separate all major tissue types. Additionally, MRI sequences cannot be used to reliably differentiate bone from the air. Therefore, most tissue-segmentation-based approaches require multiple MR sequences, which prolong image acquisition times and create a more complicated workflow^[1]^. In learning-based approaches, the features of two different domains are extracted, and then a non-linear map is generated using statistical-based learning or model fitting techniques. Huynh *et al* iteratively translated MRI patches into the related CTs by employing a random forest algorithm in conjunction with an auto-context model^[7]^. Zhong *et al* estimated pseudo-CT images from MRIs using the k-nearest neighbor regression algorithm^[8]^. They employed a supervised descriptor learning based on low-rank approximation to optimize the local descriptor of an MR image patch and to reduce its dimensionality.

Deep learning has been found to achieve the best accuracy in a number of different fields without the need for handcrafted features. This has led to the more widespread use of these networks in medical image analysis^[9–10]^. In one particular case of image synthesis, Han *et al* utilized a deep neural network that is architecturally similar to U-Net and can be trained to learn direct end-to-end mapping using MRIs with related CTs^[1]^. In a similar study, a generative adversarial network (GAN) was used to estimate CTs using MRIs and a fully convolutional network was designed which incorporated an image-gradient-difference-based loss function to avoid generating blurry target images^[11]^. Dar *et al* proposed a new deep approach for multi-contrast MRI synthesis based on conditional GAN to preserve intermediate-to-high frequency details *via* an adversarial loss^[12]^. They tried to enhance the performance with a cycle-consistency loss for unregistered images and a pixel-wise loss for registered images. Wang *et al* improved the discriminator attend for specific anatomy with an attention mechanism using selectively enhanced portions of the network during training^[13]^. Upadhyay *et al* presented an uncertainty-guided progressive learning scheme for image translation by incorporating aleatoric uncertainty as attention maps for GAN training in a progressive manner^[14]^. Dalmaz *et al* proposed a different GAN that utilized an aggregated residual transformer to combine residual convolutional and transformer modules^[15]^. They tried to promote diversity in captured representation using residual connections and distill task-relevant information using a channel compression module.

In the aforementioned methods, estimating synthetic images requires those images from the source modality and their related images in the target modality, which is referred to as paired data^[16–17]^. Providing paired data is particularly challenging because patients should undergo both MRIs and CTs and there should not be a delay between the images. Additionally, if training with unpaired data is possible, the amount of available training data increases^[17]^. Wolterink *et al* employed a GAN to be trained using unpaired CT images and MRIs^[18]^. In another study, Yang *et al* utilized a cycle generative adversarial network (CycleGAN)^[19]^ to estimate CT images using MRIs with a structure-consistency loss based on the modality of independent neighborhood descriptor^[16]^. The performance of these networks was analyzed in some specific tasks, which is not enough to demonstrate their generalizability.

In this study, a different edge-guided GAN, herein referred to as the edge-guided generative adversarial network (EGGAN) framework, is proposed for medical image-to-image translation. As paired data is difficult to obtain, we attempt to develop a framework based on unpaired data. The main contributions of this paper are as follows: (1) A different GAN based on edge detection is proposed for medical image-to-image translation using unpaired training data which utilizes an edge detector network to improve the estimated image structure; (2) A global-to-local feature fusion approach is proposed to extract and fuse local and global information progressively into the encoder-decoder deep network; (3) Network performances are assessed under different challenging medical scenarios and then compared with other deep approaches.

## Materials and methods

**Figure 1:**
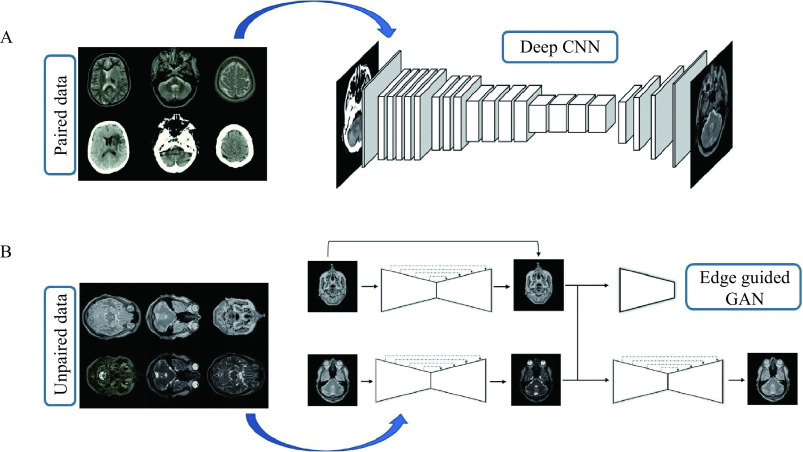
Overview of the proposed framework.

In this section, the proposed deep framework for medical image-to-image translation is presented. ***Fig. 1*** provides a visual representation of the proposed deep framework for both paired and unpaired data by an example of the CT-MRI and T2-PD translation.

### Deep neural network with global to local feature fusion

**Figure 2:**
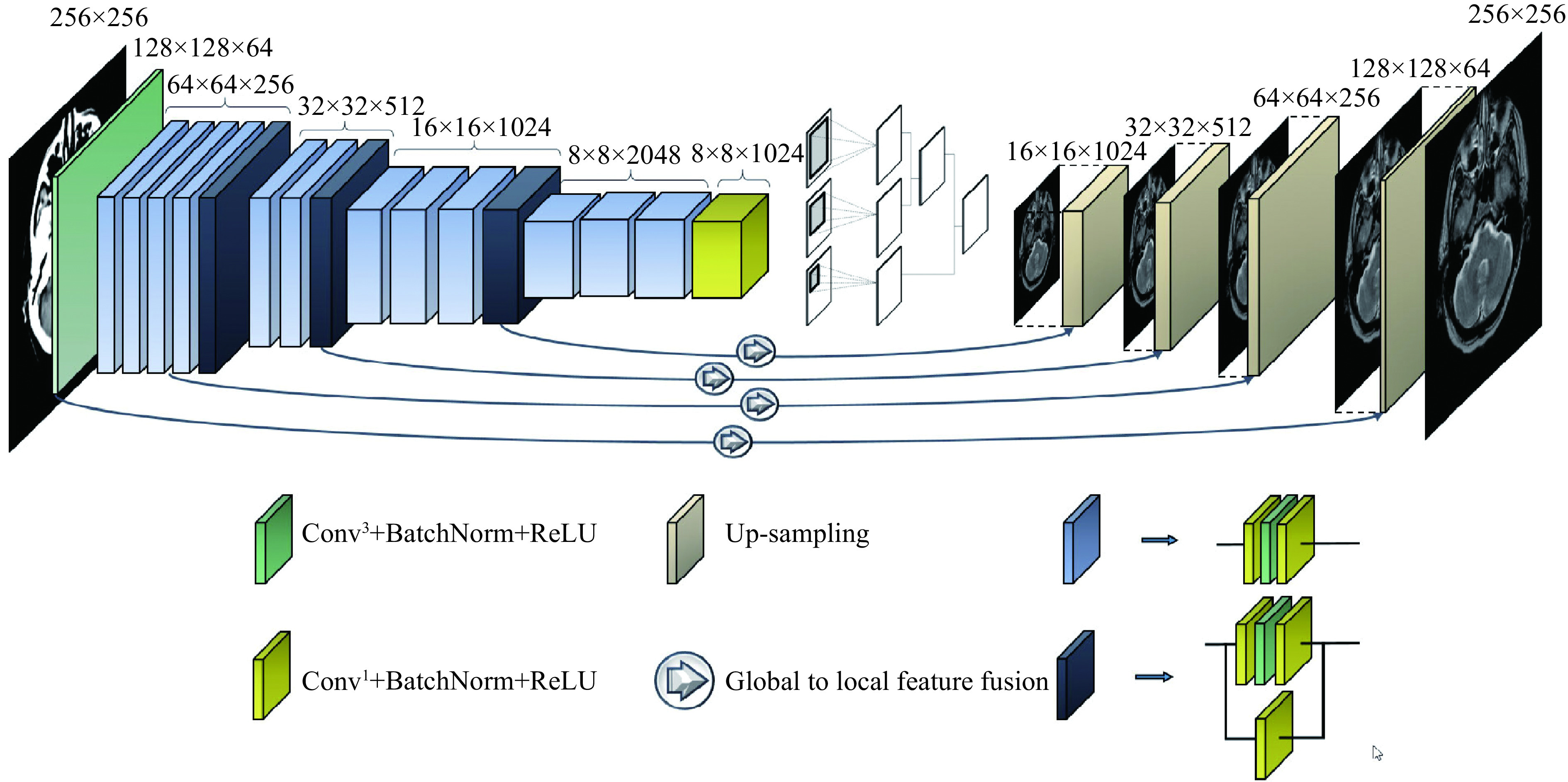
The proposed deep encoder-decoder network for image-to-image translation of medical paired data.

This network is proposed for image-to-image translation of paired data and has an encoder-decoder structure. The encoding part behaves as a traditional convolutional neural network (CNN) that learns to extract various robust and complex features using different scales from input images. In the ResNet^[10]^, the size of feature maps directly decreases to ¼ of the size after applying a pooling layer that is unsuitable, and local information of the feature maps with half the size (*i.e.*, 128×128) of the input image is required. This provides a convolutional layer in place of a pooling layer (***Fig. 2***). Fully connected layers are removed to reduce the number of tunable parameters. Accordingly, the size of generated feature maps after passing through the encoding part of the network is (8×8) (***Fig. 2***). However, the continuous residual blocks of deep learning ensure abundant features can be extracted and reduce the difficulty of training a deep network.

**Figure 3:**
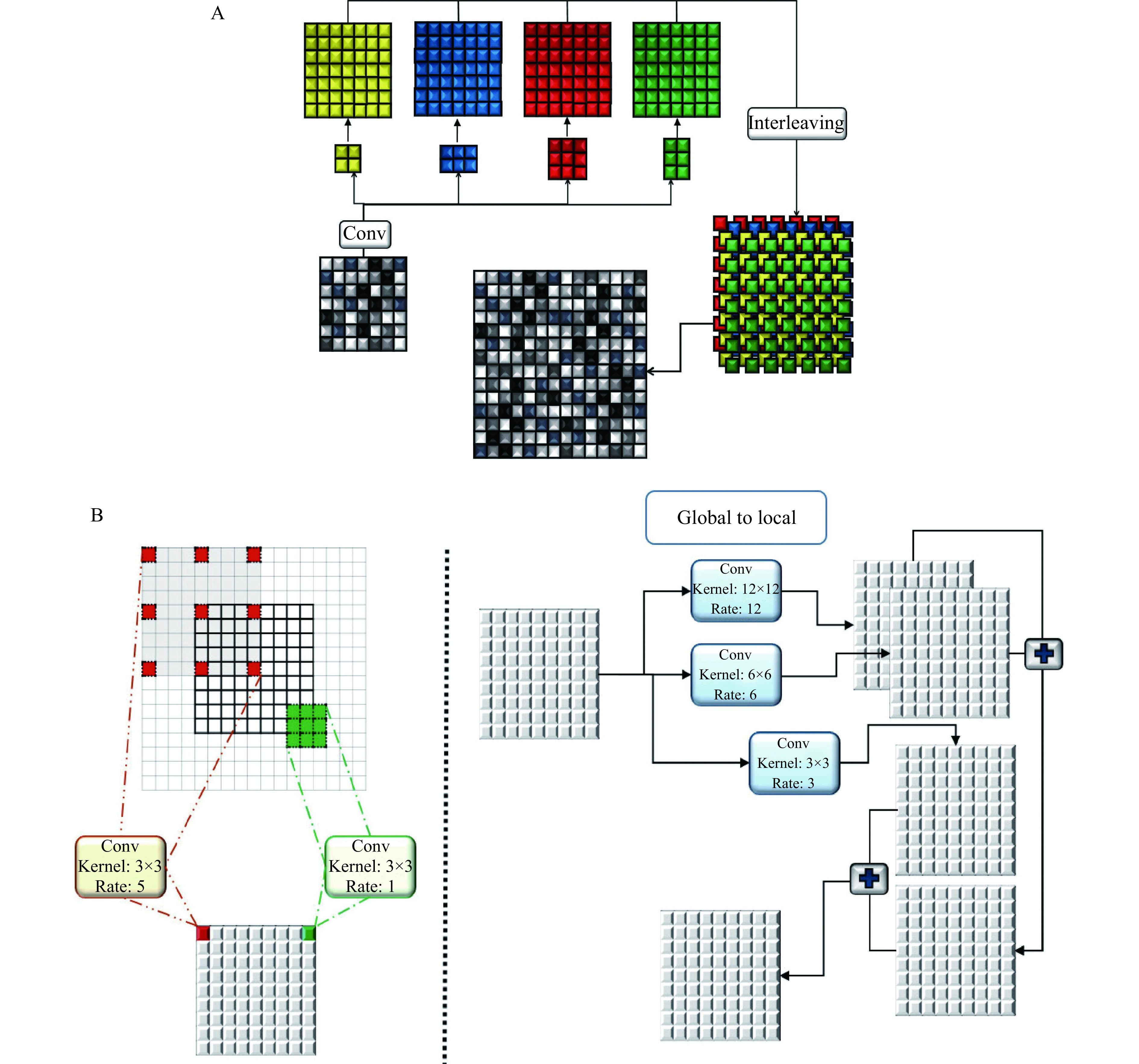
The structure of the utilized up-sampling and global to local feature fusion.

During decoding, generated coarse feature maps are transformed and the target image is gradually reconstructed from a low to high resolution. Reconstructing the target with a detailed image from the generated abstract low resolution is particularly difficult^[20]^. An up-sampling method is implemented to double the size of the feature maps at each level of the network. This method has two branches. In the first branch, four convolutional layers, 2×2, 3×2, 2×3, and 3×3, are applied to layers and results are interleaved to generate a feature map with doubled size. This procedure is repeated in the second branch with the difference that a 5×5 convolutional layer is applied. Finally, the results of the first and second layers are fused (***Fig. 3A***).

Estimating target images using an image with a different domain is a complex task that requires local and global information from the input image, simultaneously. Context information of different scales should be fused to model the dependency between different tissues and their surroundings in medical images. Hence, a global-to-local feature fusion strategy is proposed to address this issue (see ***Fig. 2***). This strategy is based on the dilated convolutional operation^[21]^, which makes it possible to expand the receptive field without increasing the number of parameters (***Fig. 3B***). The general form of discrete convolutions is defined according to equation (1):

1 $ (P{ * ^t}l)(s) = \sum\limits_{a + tb = s} {P(a)k(b),} $

where $ {{\omega}_r} = {\left[ { - r,r} \right]^2} \cap {{\text{Z}}^2} $, $ F:{{\text{Z}}^2} \to {\text{R}} $, $ l:{{\omega}_r} \to {\text{R}} $, and *t* is the dilation rate^[21]^.

In the proposed strategy, a series of dilation rates (3, 6, and 12) is selected and employed to produce multi-scale feature maps. After applying dilated convolution, the size of generated feature maps should remain unchanged, which requires carefully selecting a padding rate in accordance with the dilated rate. The higher the dilation rate, the more general the information contained will be, with wider visual cues in the produced feature maps. Therefore, generated low and high-level context information is progressively aggregated. This procedure is carried out using the highest level of context information which is produced by the largest dilated rate to the lowest-level feature maps that are produced by the smallest dilated rate (*l*_*n*_). The procedure of extracting and fusing ($ \oplus $) the feature maps (*P*) with different context information is described in equation (2):

2 $ \varphi  = P{ * ^t}{l_1} \oplus  \cdots  \oplus \left[ {P{ * ^t}{l_{n - 2}} \oplus \left[ {P{ * ^t}{l_{n - 1}} \oplus \left[ {P{ * ^t}{l_n}} \right]} \right]} \right]. $

### Proposed edge-guided GAN for unpaired data

**Figure 4:**
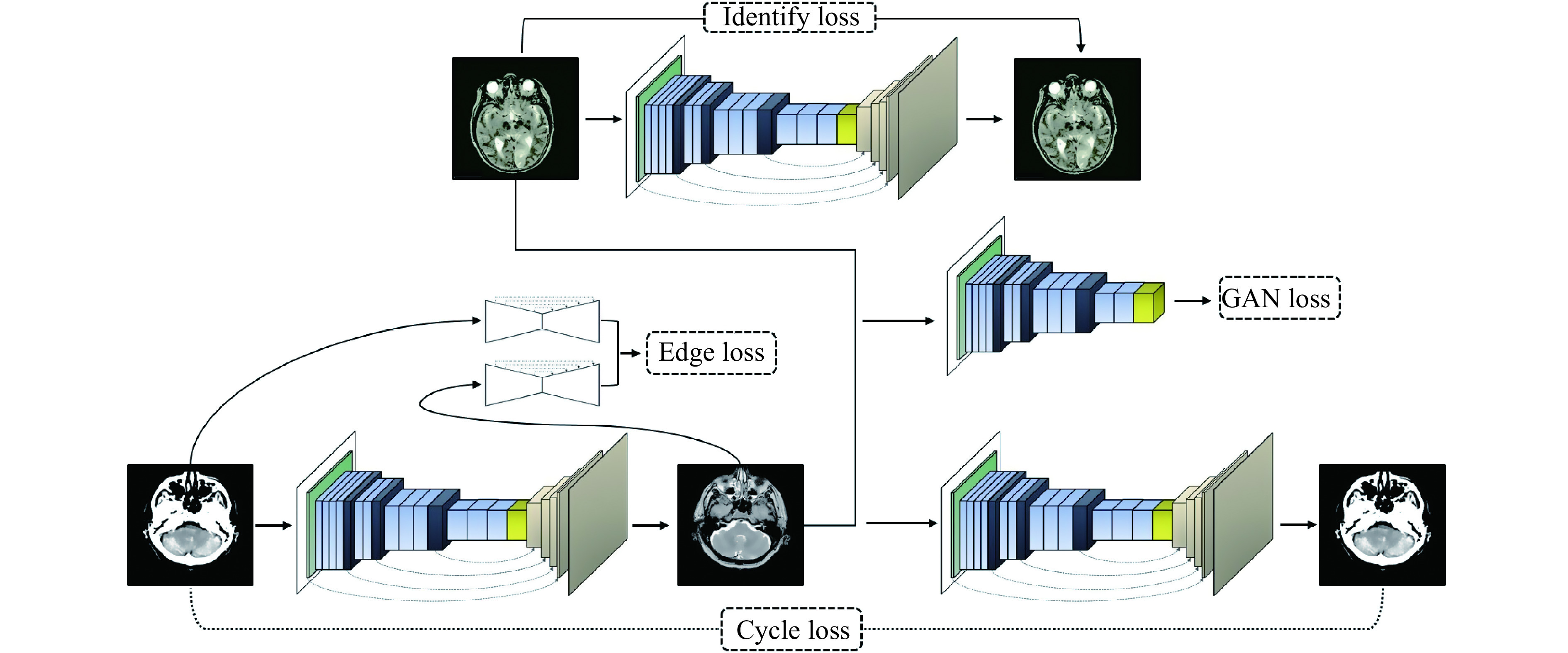
The proposed deep for image-to-image translation of unpaired medical data.

This network is proposed for image-to-image translation of unpaired data and has a structure based on the GAN. The basic structure of the proposed edge-guided GAN is represented in ***Fig. 4*** with an example of the CT-MRI translation. As shown in ***Fig. 4***, the proposed model has three main elements including the generator, edge detector, and discriminator. The generator learns to generate fake images by incorporating feedback from the discriminator. The proposed encoder-decoder network is utilized as the generator.

The discriminator tries to decide whether the results produced by the generator are considered the target. PatchGAN^[22]^ is used as a discriminator in a consistent manner with the original CycleGAN, which is a multi-layer CNN for feature extraction and classification. Edges are important parts of an image, which is vital for accurate image retrieval. Hence, the edge detection network is employed to modify structural estimates by analyzing image edges of the input domain. A network-based upon U-Net^[23]^ is trained using images and their corresponding edges, which are extracted by a canny edge detector. The trained network with fixed weights is used in the proposed EGGAN for computing the edge maintenance loss.

### Edge-based loss function

The loss function guides the training procedure of a deep neural network to obtain accurate results. This function computes the difference between real target images and approximated images^[24]^. Determining the appropriate loss function according to the desired purpose of the network is very important. Edges are crucial parts of image content, and play an important role in image reconstruction. Therefore, edges should be well-maintained to enhance the visual appearance of the image content. Hence, the loss functions of the proposed deep networks for paired and unpaired data are determined in such a way that the edges of tissue images are used in image-to-image translation as well as image reconstruction.

The loss function of the proposed deep encoder-decoder network for image-to-image translation contains three criteria, including the L2 norm (*e*_*L2*_), gradient (*e*_*G*_), and normal values (*e*_*N*_). The L2 norm is suitable for removing the overall shift in the estimated image in the target domain. The gradient and normal values are very sensitive to small shifts in tissue edges seen in medical images.

3 $ {e_{L2}} = \frac{1}{n}\sum\limits_{i = 1}^n {{{\left( {{g_e} - {g_r}} \right)}^2},} $

4 $ {e_G} = \frac{1}{n}\sum\limits_{i = 1}^n {\left[ {\left( {\left\| {{\nabla _x}({g_e}) - {\nabla _x}({g_r})} \right\| + \left\| {{\nabla _y}({g_e}) - {\nabla _y}({g_r})} \right\|} \right)} \right],} $

5 $ {e_N} = \frac{1}{n}\sum\limits_{i = 1}^n {\left[ {1 - \frac{{{n_{{g_e}}} \cdot {n_{{g_r}}}}}{{\left\| {{n_{{g_e}}}} \right\| \cdot \left\| {{n_{{g_r}}}} \right\|}}} \right],} $

6 $ {e_T} = {e_{L2}} + {e_G} + {e_N}, $

where *g*_*e*_ is the value of the estimated target image, *g*_*r*_ is the value of the reference target image, $ {\nabla _x} $is a derivation in the x-direction, $ {\nabla _y} $is a derivation in the y-direction, $ {n_{{d_e}}} $is $ \left[ {{\nabla _x}({g_e}),{\nabla _y}({g_e}),1} \right] $, $ {n_{{d_r}}} $is $ \left[ {{\nabla _x}({g_r}),{\nabla _y}({g_r}),1} \right] $, and *n* is the total number of pixels.

The loss function of the proposed network for image-to-image translations of paired data contains two criteria, including edge maintenance and CycleGAN losses. The edge detector network (*F*) is used to extract the edge of the estimated images and reference images. Edge maintenance loss (*e*_*EM*_) is computed as the difference between the edges of the estimated images and reference images:

7 $ {e_{EM}} = \frac{1}{n}\sum\limits_{l = 1}^n {\left( {\left\| {F({g_r}) - F({g_r})} \right\| + \left\| {F({g_e}) - F({g_r})} \right\|} \right).} $

CycleGAN loss has three components, including cycle-consistency loss, GAN loss, and identity loss^[19]^. Identity loss is used to regulate the generator in order to avoid superfluous translation and ensure results are closer to the identity translation. Cycle-consistency loss constraints the mapping function space and makes it possible for the generator to decouple the style and content of input images. Additionally, cycle-consistency permission training is used for the proposed EGGAN, with unpaired data. The LSGAN^[25]^ is used as the GAN loss.

8 $ {e_{CG}} = {e_{{\text{LSGA}}{{\text{N}}_1}}} + {e_{{\text{LSGA}}{{\text{N}}_2}}} + {\lambda _{Cyc}}{e_{Cyc}} + {\lambda _I}{e_{iden}}, $

9 $ {e_T} = {e_{CG}} + {\lambda _{EM}}{e_{EM}}, $

where *e*_*CG*_ is the CycleGAN loss; *e*_*Cyc*_ and *e*_*iden*_ are the cycle-consistency and identity losses, respectively; *λ*_*Cyc*_ and *λ*_*iden*_ are the weights of the cycle-consistency and identity losses, respectively; and *λ*_*EM*_ is the weight of the edge maintenance loss.

### Dataset

Two datasets are utilized in this study, both of which have CT-MRI and PD-T2 paired data. These were used to analyze the proposed encoder-decoder network for paired data and the proposed EGGAN for unpaired data.

#### MRI-CT dataset

This dataset contains MRIs from 18 patients who were randomly selected. MRIs were obtained using a 1.5 T Siemens Avanto scanner using a T1-weighted 3D spoiled gradient (repetition time 11 milliseconds, echo time 4.6 milliseconds, field-of-view 256×256×160 mm^3^, and flip angle 20°). Related CT images were obtained using a Siemens Sensation 16 scanner with tube voltage 120 kV, in-plane resolution 0.5×0.5 mm^2^, exposure 300 mAs, and slice thickness of 1 mm (https://github.com/ChengBinJin/MRI-to-CT-DCNN-TensorFlow).

#### IXI dataset

**Figure 5:**
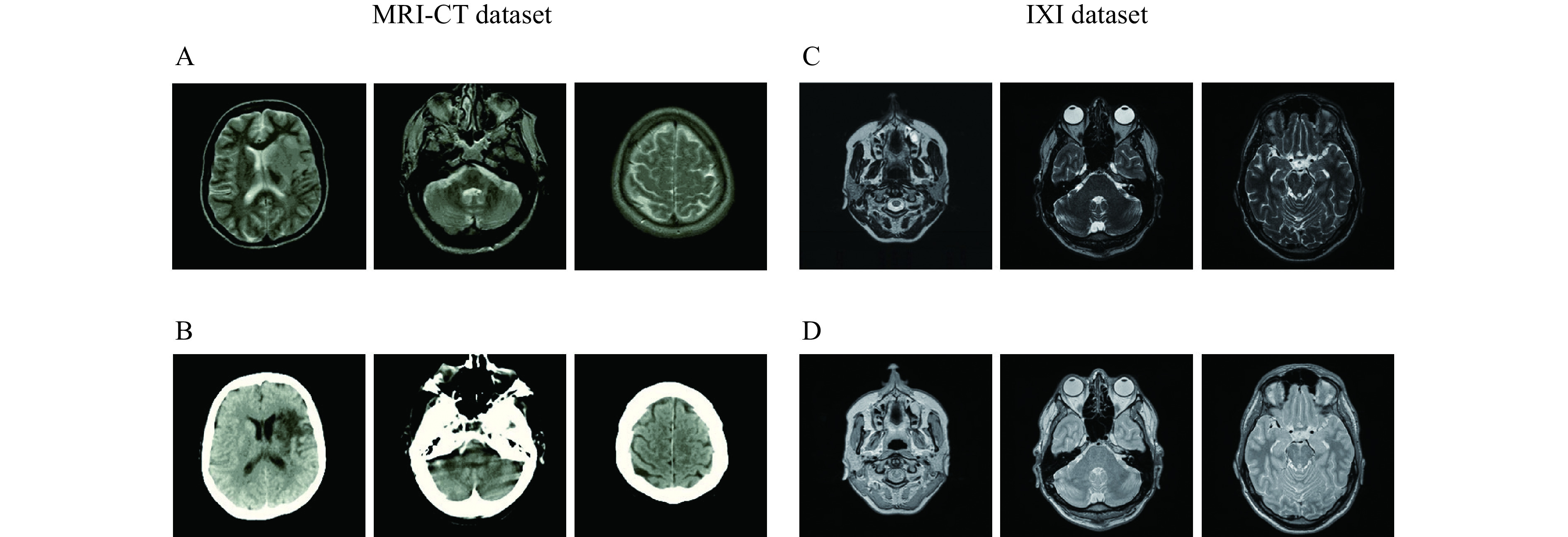
Certain samples of the utilized dataset for implementing the proposed encoder-decoder network.

This dataset contains nearly 600 MRIs from relatively healthy participants, which were collected by three different hospitals in London including the Hammersmith Hospital which used a Philips 3T system, Guy's Hospital which used a Philips 1.5T system, and the Institute of Psychiatry which used a GE 1.5T system. PD and T2 images obtained using the Philips Medical Systems Gyroscan Intera 1.5T have the following parameters: repetition time 8178 milliseconds, echo train length 16, number of phase encoding steps 187, and flip angle 90° (http://brain-development.org/ixi-dataset). PD and T2 images obtained using the Philips Medical Systems Intera 3T have the following parameters: repetition time 5725 milliseconds, echo train length 16, number of phase encoding steps 187, acquisition matrix 192×187, and flip angle 90°. ***Fig. 5*** represents certain samples of the utilized datasets.

## Results

In this section, an assessment of results from the proposed image-to-image translation are reported. Computations have been carried out using a single NVIDIA GeForce GTX 1080 Ti with 11 GB of GPU memory.

### Quality assessment

Criteria used for analyzing the performance of the proposed deep networks are defined in this section. There is no consensus within the scientific community regarding criteria for assessing the performance of the models studied here^[26]^. Therefore, four different criteria including peak-signal-to-noise-ratio (*E*_*PSNR*_)^[27]^, mean absolute error (*E*_*MAE*_), root-mean-squared error (*E*_*RMSE*_), and structural similarity index measure (*E*_*SSIM*_)^[28]^ were used, and were computed by comparing the estimated target image values with corresponding values in the reference data^[18,29]^.

10 $ {E_{RMS E}} = \sqrt {\frac{1}{n}\sum\limits_{i = 1}^n {{{\left( {{g_e} - {g_r}} \right)}^2}} } , $

11 $ {E_{MAE}} = \frac{1}{n}\sqrt {\sum\limits_{i = 1}^n {\left\| {{g_e} - {g_r}} \right\|} } , $

12 $ {E_{PS NR}} = 20{\log _{10}}\frac{{4\;095}}{{\dfrac{1}{n}\displaystyle\sum\limits_{i = 1}^n {{{\left( {{g_e} - {g_r}} \right)}^2}} }}, $

13 $ {E_{S S IM}}({g_e},{g_r}) = \frac{{\left( {2{\mu _{{g_e}}}{\mu _{{g_r}}} + {c_1}} \right)\left( {2{\sigma _{{g_e}{g_r}}} + {c_2}} \right)}}{{\left( {\mu _{{g_e}}^2 + \mu _{{g_r}}^2 + {c_1}} \right)\left( {\sigma _{{g_e}}^2 + \sigma _{{g_r}}^2 + {c_2}} \right)}}, $

where *μ* and *σ* are the mean and variance of the considered variable.

### Paired image-to-image translation

In this section, the results from the proposed encoder-decoder network under different scenarios are presented. Four challenging image-to-image translations including CT-MRI, MRI-CT, T2-PD, and PD-T2 are selected. In the T2-PD and PD-T2 translation, the imaging modality is the same, and the acquired contrast values are different because of the procedure of data collection. At the same time, in the CT-MRI and MRI-CT translations, the imaging modality of the data is different, which makes the issue more difficult and challenging. Additionally, the proposed network is utilized for eliminating motion effects from the medical MR images and translating these into corresponding motion-free images.

In the CT-MRI and MRI-CT translation, images from 9 patients are selected as training data and images of the remaining 9 patients are selected as test data. As each image volume has approximately 150 slices, approximately 2700 training and testing samples are provided.

In the PD-T2 and T2-PD translation, images from 60 patients are employed and half of those are used as training data while the remaining thirty were selected as test data. These images have approximately 130 slices, resulting in 7800 training and testing samples.

In the motion correction procedure, T1 images from 40 patients are utilized and the motion artifacts are generated upon those. After which, the provided paired images are divided into two same-size groups for both training and testing. As the T1 image has approximately 150 slices, approximately 6000 samples are provided for implementation.

All the provided images are resampled to 256×256 pixels and the following data augmentation was applied to all datasets.

• Translation: the batch data are horizontally and vertically flipped with 0.5 probability;

• Flip: a certain part of the batch data is randomly cropped;

• Rotation: the batch data are rotated by $ \theta  \in [ - {5^ \circ },{5^ \circ }] $

**Figure 6:**
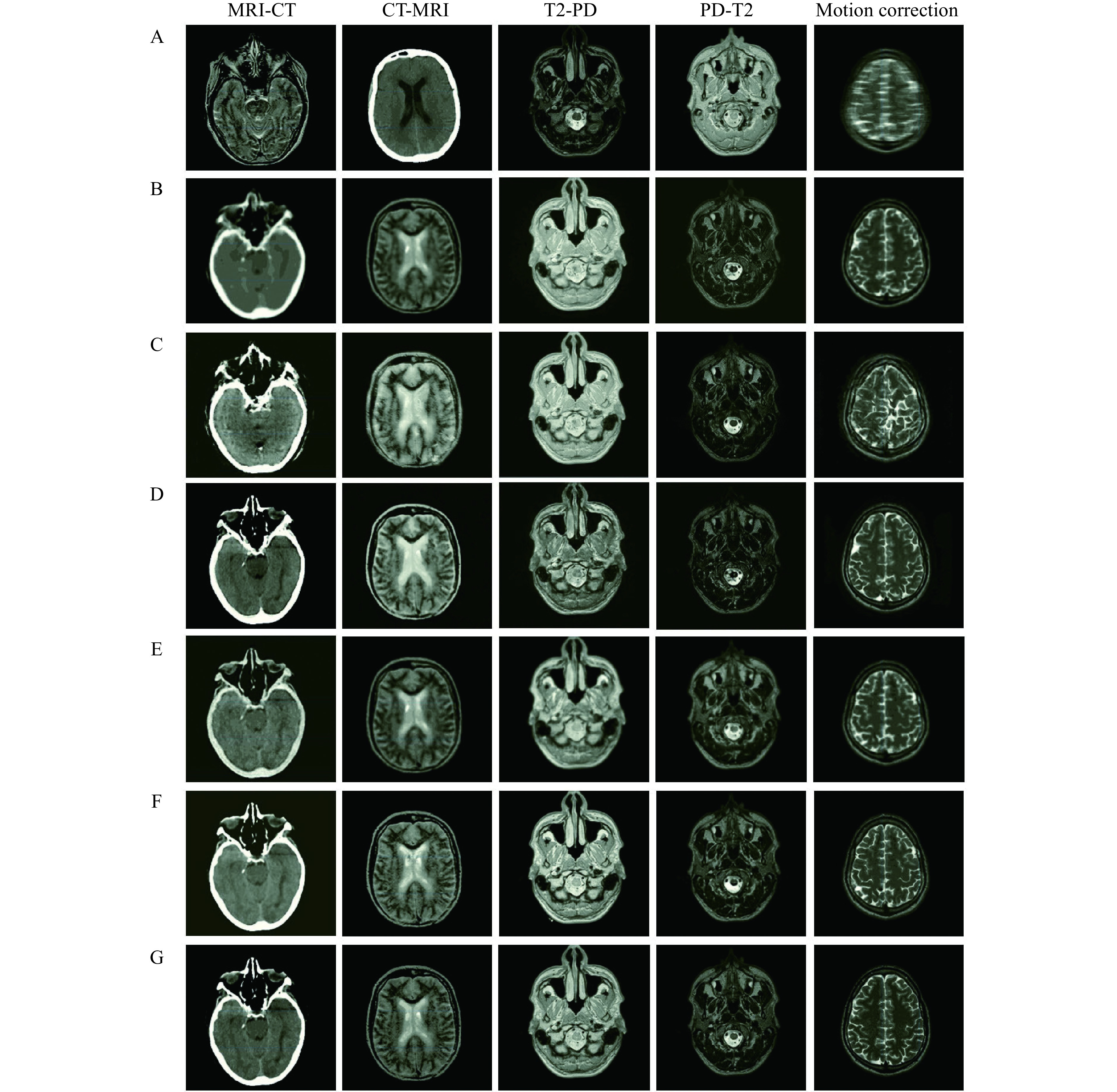
Visual comparison of the proposed encoder-decoder network for paired image-to-image translation with some commonly used deep networks in the designed tasks.

The number of epochs, batch size, momentum, and learning rate for the proposed encoder-decoder network in these tasks are considered to be 100, 2, 0.9, and 10^−4^, respectively. The performance of the proposed network is evaluated and compared with several well-known networks including CycleGAN^[19]^, uncertainty-guided progressive generative adversarial network (UPGAN)^[14]^, registration generative adversarial network (RegGAN)^[30]^, and Pix2Pix^[22]^. It should be noted that the number of epochs are learning rate are selected by cross validation. The performance is analyzed on the five mentioned tasks (MRI-CT, CT-MRI, T2-PD, PD-T2, and Motion correction). In this regard, different numbers of epoch in the set (20, 40, ···, 100) and learning rates in the set (10^−5^, 5×10^−^^5^, 10^−4^, 5×10^−4^, 10^−3^) are investigated. The result of image-to-image translations using the proposed network and other mentioned networks in different tasks is represented in ***Fig. 6***. The results of the mentioned criteria are reported in ***Table 1***.

According to ***Fig. 6***, the edges and structure of the obtained results using the Pix2Pix and CycleGAN networks are different from the reference data, which indicates an inability of these networks to retrieve boundary and structural information. Additionally, the achieved intensity values using CycleGAN and RegGAN are very different from reference data. Although the obtained results of UPGAN are relatively suitable for different tasks, their quality and sharpness are lower than those in the proposed network. Employing the global-to-local feature fusion strategy significantly improves the structure of results. Using a loss function also emphasizes edges which improves the sharpness and quality of tissue boundaries (***Fig. 6***).

The evaluation (***Table 1***) highlighted a better performance of the proposed encoder-decoder network across all criteria. Of course, during image translation MRI-CT and CT-MRI, inputs and outputs belong to different domains. However, for T2-PD and PD-T2, the inputs and outputs belong to the same domain and the recorded intensity values are different. The translation between two different domains (MRI-CT and CT-MRI) is much more challenging than the conversion of intensity values used in T2-PD and PD-T2. The effect of this can be observed in the results of the UPGAN, CycleGAN, RegGAN, and Pix2Pix modeling (***Table 1***).

In the results obtained from the proposed network, translations between two different domains are not significantly reduced compared to the translation of intensity values. This is due to the power structure of the proposed network in feature extraction, feature integration, and data retrieval. It should be noted that the higher the values for *E*_*RMSE*_ and *E*_*MAE*_, the greater the error of the obtained results. Likewise, the higher the values for *E*_*PSNR*_ and *E*_*SSIM*_, the greater the accuracy of the results obtained.

### Unpaired image-to-image translation

In this section, the performance of the proposed EGGAN is evaluated for image-to-image translation using unpaired data. The aforementioned scenarios were re-implemented using unpaired data. The selected training and test data for different scenarios in the previous section are utilized. Only target images are shuffled so that they are not paired with input images. The number of epochs, batch size, momentum, and learning rate for the proposed EGGAN in these tasks are considered to be 100, 4, 0.9, and 5×10^−4^, respectively. It should be noted that the number of epochs, learning rate, *λ*_*Cyc*_, *λ*_*iden*_, and *λ*_*EM*_ are selected by cross validation. The number of epochs and learning rate are specified the same as paired translation.

**Figure 7:**
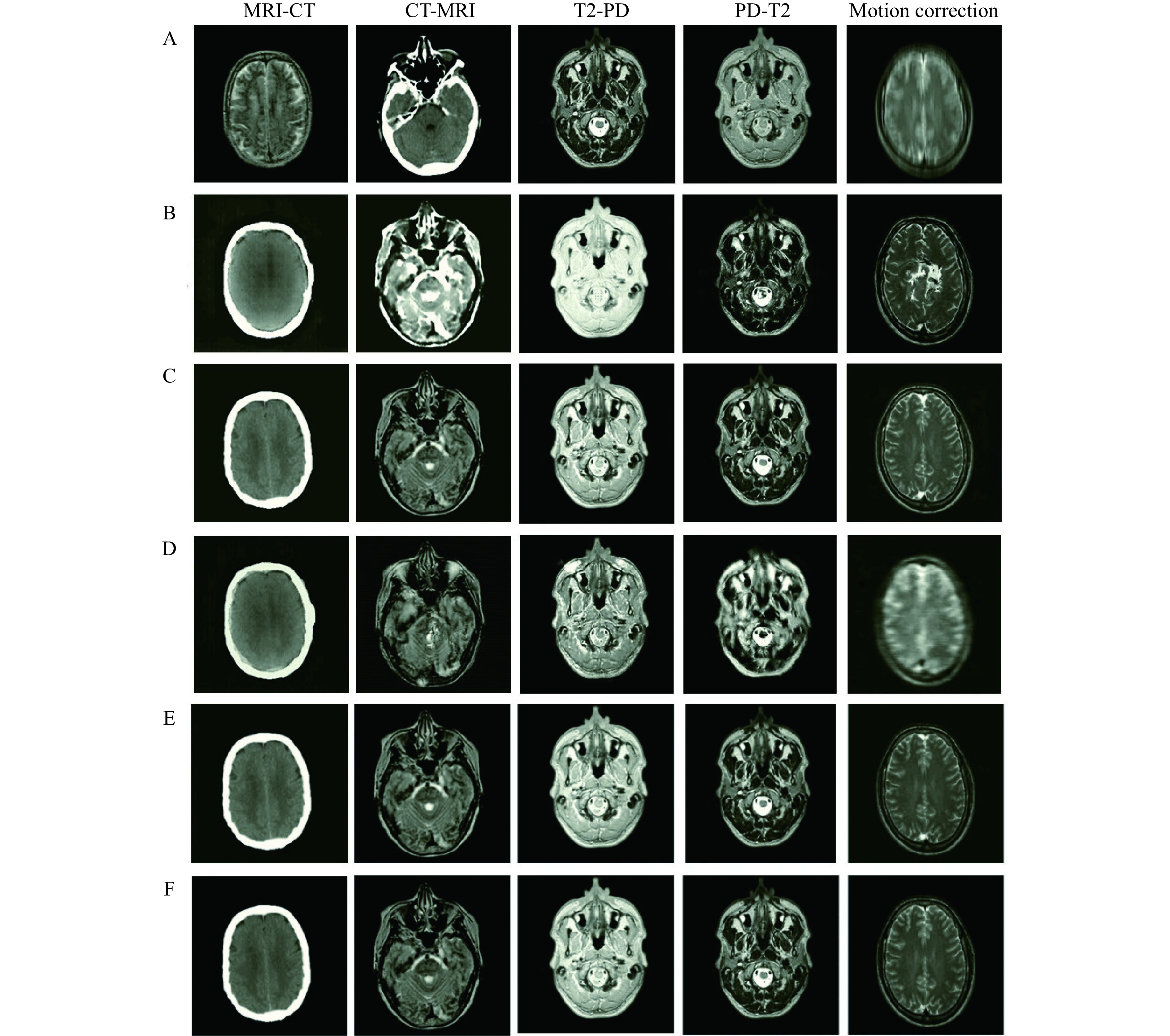
Visual comparison of the proposed edge-guided generative adversarial network for unpaired image-to-image translation with some common deep networks in the designed tasks.

Different *λ*_*Cyc*_, *λ*_*iden*_, and *λ*_*EM*_ in the set [1, 2, 5, 10, 20] are analyzed, and *λ*_*Cyc*_=10, *λ*_*iden*_=10, and *λ*_*EM*_=5 are determined for all experiments. As in the previous section for paired analysis, the performance of the proposed EGGAN was evaluated and compared with several well-known networks dealing with unpaired data including, CycleGAN^[19]^, AGAN^[31]^, and RegGAN^[30]^. Certain samples of image-to-image translation of unpaired data are presented in ***Fig. 7*** and the quantitative evaluation is reported in ***Table 2***.

According to ***Fig. 7***, one can see that proper edge retrieval is a major problem for image translation using unpaired training data. This increases the error of CycleGAN, AGAN, and RegGAN networks, especially in motion correction where edges are distorted, and accurate reconstruction of the edges is very important. This also occurs in translation between two different domains (MRI-CT and CT-MRI), where the boundaries of the target should be estimated. An attempt was made to overcome these issues by employing the proposed encoder-decoder network as the generator and using an edge-based loss function to estimate accurate results and preserve the tissue boundaries.

Our evaluation (***Table 2***) demonstrates that the proposed EGGAN outperforms other networks. Although unpaired training data was used, the proposed network is still successful in estimating the values. As a result, by using the EGGAN, high accuracy can be achieved if there is no available paired data.

**Figure 8:**
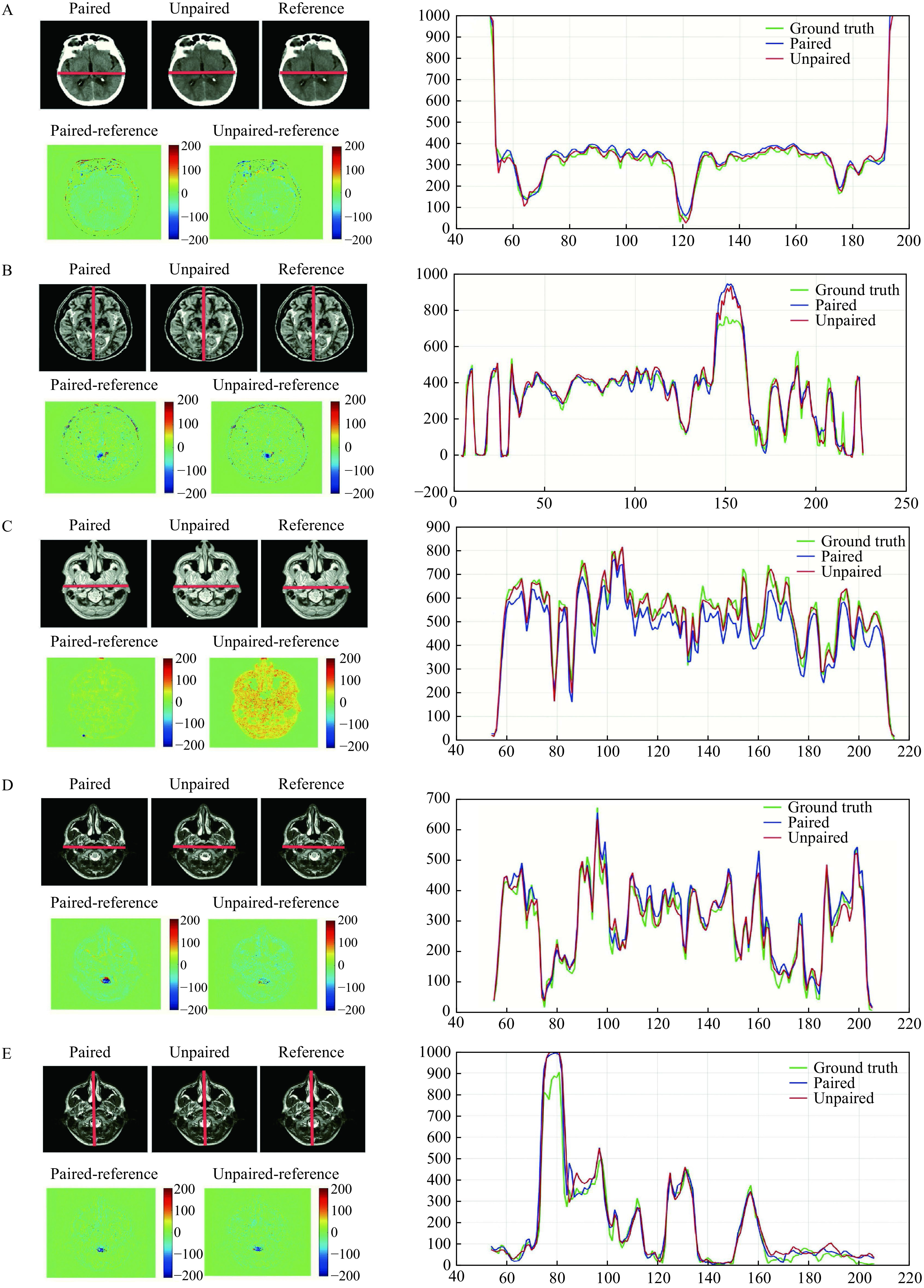
Comparison of the proposed network for paired and unpaired image-to-image translation in the designed tasks.

The quantitative analysis of the trained network with paired and unpaired data performed similarly well (***Table 1*** and ***2***). For the visual analysis, the results of the trained encoder-decoder network by paired training data and the trained EGGAN by unpaired training data are compared in ***Fig. 8***. This figure exhibits a 1D profile passing through three red lines including the ground truth data, images estimated using the encoder-decoder network trained by paired data, and images estimated using the EGGAN trained by unpaired data. A different image was computed by subtracting the ground truth images and estimated images. A comparison of the 1D profiles and an analysis of their ascent and descent trends show great closeness and similarity of the translated images using the trained networks by paired and unpaired data. The generated difference maps show slight errors in some parts for the estimated images.

## Discussion

In this study which included a comprehensive evaluation of the proposed framework, three different image-to-image translation scenarios were designed and implemented. In the first scenario, the goal was to translate MRI products, *i.e.*, PD to T2 and T2 to PD. In this scenario, the geometry and structure of input and output images were the same. Yet, intensity values were different owing to differences in the way the images were provided. In the second scenario, the goal was to translate input images to images of a different domain, *i.e.*, MRI to CR and CT to MRI. In this scenario, in addition to the intensity values in the two images, the geometric structure of the images is also different, which poses a more substantial challenge. In the third scenario, the goal was to eliminate errors created during imaging, *e.g.*, the motion artifact. In this case, the intensity values were relatively similar, but the geometry of the image has changed due to errors.

The performances of the proposed networks for paired and unpaired training data were evaluated quantitatively (***Fig. 6*** and ***7*** as well as ***Table 1*** and ***2***). According to the achieved *E*_*MAE*_, *E*_*RMSE*_, *E*_*PNSR*_, and *E*_*SSIM*_ (***Table 1*** and ***2***), the proposed encoder-decoder network for paired training data and the proposed EGGAN for unpaired training data achieved the best performance against the other networks in all the designed translation tasks. This may be partially due to the fact that some hidden details of the input images were unrecognizable to the generators of these models and produce some fake details to match their loss functions. Hence, these fake details reduce their accuracy and make the results worse than the results of the EGGAN. Indeed, in the generator for the EGGAN model, the details are extracted using the proposed global to local feature fusion strategy.

According to the difference map of the ground truth and the corresponding estimated images (***Fig. 8***), the errors in the CT-MRI and MRI-CT translations are the outer boundaries of the head area. This may be partially attributed to imperfect alignment between the MRI and CT images. In the other translations (PD-T2, T2-PD, and motion correction), the proposed networks with edge-based loss learn to distinguish different anatomical structures in the head area from similar input pixel values. It is challenging to obtain correspondence pixels between the MRIs and CT images by a linear registration^[1]^. This causes inaccuracies in the model during the training procedure. The cross-modality registration problem is converted into a single modality registration problem by converting the CT-MRI and MRI-CT in which the inputs and outputs belong to different modalities.

Most medical image translation methods use paired data for network training^[11–12,15,32–36]^, and a limited number of methods focus on using unpaired data^[16,37]^. One of the strengths of the proposed deep framework is to present a network capable of training using unpaired data for medical image translation. In addition, the performance of the proposed network was examined and analyzed in different scenarios. Research in this field, especially research related to image translation using unpaired data, a specific translation (MRI to CT) has been considered^[16,37]^. Additionally, in the proposed deep neural network an edge-guided strategy was considered to improve the accuracy of the achieved results, especially for tissue boundaries. This is very important for diagnosis, although in most of the existing methods simple U-Net and GAN are used without considering edge information^[38–42]^. Finally, a global-to-local feature fusion strategy was proposed instead of using simple skip connections^[1,38–39,41,43–44]^. This enabled us to extract various robust features through down-sampling as well as to used these during the up-sampling procedure. Investigating the 3D architecture of deep neural networks for the analysis of 3D multi-channel volumes will be suggested for future work.

**Table 1:** Performance analysis and quantitative comparison of the proposed encoder-decoder network for paired image-to-image translation with some common deep networks in the designed tasks

	Method	*E*_*RMSE*_	*E*_*MAE*_	*E*_*PSNR*_	*E*_*SSIM*_
MRI-CT	Pix2Pix^[22]^	47.37	21.24	18.02	0.70
	CycleGAN^[19]^	44.49	20.70	17.97	0.71
	RegGAN^[30]^	36.34	18.53	19.24	0.78
	UPGAN^[14]^	25.34	12.36	21.10	0.85
	Proposed deep network	**19.48**	**7.64**	**22.56**	**0. 94**
CT-MRI	Pix2Pix^[22]^	54.24	23.51	17.81	0.73
	CycleGAN^[19]^	47.25	22.40	18.45	0.74
	RegGAN^[30]^	39.33	15.84	19.74	0.79
	UPGAN^[14]^	31.91	12.18	20.12	0.81
	Proposed deep network	**20.42**	**9.20**	**22.36**	**0.96**
T2-PD	Pix2Pix^[22]^	51.34	19.41	18.94	0.81
	CycleGAN^[19]^	58.47	22.98	16.20	0.85
	RegGAN^[30]^	41.49	17.16	19.91	0.89
	UPGAN^[14]^	30.45	14.01	22.01	0.92
	Proposed deep network	**26.35**	**11.63**	**23.01**	**0.92**
PD-T2	Pix2Pix^[22]^	44.21	19.34	18.12	0.79
	CycleGAN^[19]^	51.10	21.11	17.24	0.74
	RegGAN^[30]^	32.94	14.35	19.04	0.81
	UPGAN^[14]^	23.56	10.41	21.05	0.88
	Proposed deep network	**19.13**	**8.89**	**23.38**	**0.90**
Motion correction	Pix2Pix^[22]^	42.06	18.24	18.94	0.77
	CycleGAN^[19]^	45.12	17.34	17.63	0.71
	RegGAN^[30]^	35.25	14.25	17.75	0.74
	UPGAN^[14]^	34.36	13.75	18.87	0.81
	Proposed deep network	**22.13**	**9.65**	**21.91**	**0.90**
The best results are bold in the table. *E*_*RMSE*_: root mean squared error, *E*_*MAE*_: mean square, *E*_*PNSR*_: error, peak-signal-to-noise-ratio, *E*_*SSIM*_: structural similarity index measure. MRI-CT: magnetic resonance imaging-computed tomography; CT-MRI: computed tomography-magnetic resonance imaging; PD: proton density; CycleGAN: cycle generative adversarial network; RegGAN: registration generative adversarial network; AGAN: attention generative adversarial network; UPGAN: uncertainty-guided progressive generative adversarial network.					

**Table 2:** Performance analysis and quantitative comparison of the proposed EGGAN for unpaired image-to-image translation with some common deep networks in the designed tasks

	Method	*E*_*RMSE*_	*E*_*MAE*_	*E*_*PSNR*_	*E*_*SSIM*_
MRI-CT	CycleGAN^[19]^	120.39	62.85	9.35	0.65
	AGAN^[31]^	54.53	23.49	14.62	0.86
	RegGAN^[30]^	27.14	8.68	20.02	0.93
	Proposed deep network	**21.54**	**8.12**	**21.44**	**0.94**
CT-MRI	CycleGAN^[19]^	85.41	42.84	10.72	0.54
	AGAN^[31]^	48.94	19.36	17.78	0.48
	RegGAN^[30]^	22.25	10.20	21.62	0.95
	Proposed deep network	**20.01**	**9.53**	**22.51**	**0.96**
T2-PD	CycleGAN^[19]^	93.07	51.03	9.14	0.75
	AGAN^[31]^	56.23	26.90	14.60	0.78
	RegGAN^[30]^	55.08	26.10	14.77	0.80
	Proposed deep network	**34.61**	**15.30**	**19.70**	**0.93**
PD-T2	CycleGAN^[19]^	66.51	36.42	12.14	0.52
	AGAN^[31]^	48.85	21.68	15.08	0.72
	RegGAN^[30]^	44.06	19.05	16.06	0.79
	Proposed deep network	**26.09**	**10.90**	**20.34**	**0.92**
Motion correction	CycleGAN^[19]^	77.74	36.18	10.70	0.43
	AGAN^[31]^	37.55	17.20	17.52	0.76
	RegGAN^[30]^	33.80	15.10	18.21	0.84
	Proposed deep network	**22.32**	**9.85**	**21.86**	**0. 90**
The best results are bold in the table. *E*_*RMSE*_: root mean squared error, *E*_*MAE*_: mean square, *E*_*PNSR*_: error, peak-signal-to-noise-ratio, *E*_*SSIM*_: structural similarity index measure. MRI-CT: magnetic resonance imaging-computed tomography; CT-MRI: computed tomography-magnetic resonance imaging; PD: proton density; CycleGAN: cycle generative adversarial network; RegGAN: registration generative adversarial network; AGAN: attention generative adversarial network; UPGAN: uncertainty-guided progressive generative adversarial network.					
